# Expression patterns of programmed death ligand 1 correlate with different microenvironments and patient prognosis in hepatocellular carcinoma

**DOI:** 10.1038/s41416-018-0144-4

**Published:** 2018-06-20

**Authors:** Chao-Qun Liu, Jing Xu, Zhong-Guo Zhou, Li-Lian Jin, Xing-Juan Yu, Gang Xiao, Jie Lin, Shi-Mei Zhuang, Yao-Jun Zhang, Limin Zheng

**Affiliations:** 1 Collaborative Innovation Center for Cancer Medicine, State Key Laboratory of Oncology in South China, Sun Yat-sen University Cancer Center, Guangzhou, 510060 P. R. China; 2 Department of Hepatobiliary Oncology, Sun Yat-sen University Cancer Center, 510060 Guangzhou, P. R. China; 3 Key Laboratory of Gene Engineering of the Ministry of Education, State Key Laboratory of Biocontrol, School of Life Sciences, Sun Yat-sen University, Guangzhou, 510275 P. R. China

**Keywords:** Prognostic markers, Tumour biomarkers, Cancer microenvironment, Tumour biomarkers, Cancer

## Abstract

**Background:**

Recent clinical studies have suggested that programmed death ligand 1 (PD-L1) expression in a tumour could be a potential biomarker for PD-L1/PD-1 blockade therapies.

**Methods:**

To better characterise PD-L1 expression in hepatocellular carcinoma (HCC), we analysed its expression patterns in 453 HCC patients by double staining for CD68 and PD-L1 using the Tyramide Signal Amplification Systems combined with immunohistochemistry. We also investigated its correlation with clinical features, prognosis and immune status.

**Results:**

The results showed that PD-L1 expression on tumour cells (TCs) was negatively associated with patients’ overall survival (OS; *P* = 0.001) and relapse-free survival (RFS; *P* = 0.006); however, PD-L1 expression on macrophages (Mφs) was positively correlated with OS (*P* = 0.017). Multivariate analysis revealed that PD-L1 expression on TCs and Mφs were both independent prognostic factors for OS (hazard ratio (HR) = 1.168, *P* = 0.004 for TC-PD-L1; HR = 0.708, *P* = 0.003 for Mφ-PD-L1). Further studies showed that Mφ-PD-L1^+^ tumours exhibited an activated immune microenvironment, with high levels of CD8^+^ T-cell infiltration and immune-related gene expression.

**Conclusion:**

Our study provided a novel methodology to evaluate PD-L1 expression in the tumour microenvironment, which might help to select patients who would benefit from anti-PD-1/PD-L1 immunotherapies.

## INTRODUCTION

Hepatocellular carcinoma (HCC) is one of the most prevalent tumours, with a high disease incidence and high mortality worldwide.^1^ Roughly one third of patients are eligible for potential curative treatments, such as surgical resection, radiofrequency ablation, and liver transplantation.^2^ However, these therapeutic treatments often do not provide a complete cure, and half of the treated patients develop tumour recurrence within 3 years.^3–5^ Although adjuvant therapies, including transarterial chemoembolisation and adoptive transfer of cytokine-induced killer cells, can improve the overall survival (OS) in some patients with HCC, their effects on tumour regression remain limited.^6,7^

Immune checkpoint blockade therapy, which targets regulatory pathways in T cells to unleash immunosuppression and restore antitumour immune responses, has been hailed as a major breakthrough in oncology and provides a new weapon against cancer.^8,9^ Agents targeting the programmed death ligand 1 (PD-L1)/programmed cell death protein-1 (PD-1) immune checkpoint displayed impressive antitumour effects and durable responses on a fraction of patients with melanoma, bladder carcinoma, non-small-cell lung carcinoma, Hodgkin’s lymphoma, and renal cell carcinoma.^10–14^ Durable responses have been reported recently in patients with advanced HCC treated with Nivolumab, a fully human immunoglobulin antibody that disrupts PD-1 immune checkpoint signalling. In this multicentre phase 1/2 study, treatment of patients with advanced HCC with Nivolumab resulted in substantial tumour reductions and objective response rates of 15–20%, irrespective of the line of therapy.^15^ Despite these encouraging results, a major challenge is to identify biomarkers that predict therapeutic responsiveness, which could be used to select patients who might benefit from the treatment.

Although PD-L1 is not yet established as a consistently reliable biomarker across tumour types, emerging evidence indicates that patients who have a higher proportion of tumour cells (TCs) or immune cells-expressing PD-L1 might achieve greater benefit.^16–18^ PD-L1 can be constitutively or inducibly expressed on both TCs and immune cells in the tumour microenvironment (TME).^8,19,20^ Several clinical studies have observed that patients with PD-L1^+^ TCs were more likely to benefit from checkpoint blockade therapies^21,22^; while some studies emphasised that immune cells-expressing PD-L1 correlated with patients’ responses.^16,23,24^ Based on these findings, the US Food and Drug Administration has approved several PD-L1 assays to detect the overall PD-L1 expression level as a criterion to select patients for immunotherapy with anti-PD-1/PD-L1 antibodies. However, the levels of overall PD-L1 expression did not yield satisfactory results as a predictive biomarker to select patients who would benefit from treatment in clinical practice.^25^ Therefore, it is essential to optimise the detection method and understand the relevance of PD-L1 expression patterns in tumour tissues.

In the present study, we aimed to characterise PD-L1-expression in patients with HCC and investigate the correlation between the expression patterns of PD-L1 and patients’ clinical features and prognosis. The TME features of different PD-L1 expression patterns were further explored using the Agilent human expression microarray to reveal their potential biological and clinical significance.

## Materials and methods

### Patients

All samples were anonymously coded in accordance with local ethical guidelines (as requested by the Declaration of Helsinki), with written informed consent and a protocol approved by the Review Board of Sun Yat-sen University Cancer Center.

Formalin-fixed, paraffin-embedded tissue from 453 patients with pathologically confirmed HCC, who had all received resection for tumours at the Sun Yat-sen University Cancer Center between 2006 and 2010, were enrolled in this study. The inclusion criteria used in patient enrolment were: Absence of anticancer therapies or distant metastasis before surgery; no concurrent autoimmune disease, HIV or syphilis; and availability of follow-up data. All patients underwent curative resection for HCC with the following intra-operative goals: A resection margin of at least 1 cm, complete resection of all tumour nodules, and leaving the cut surface free of tumour (based on histological examination). Intra-operative ultrasound and post-surgical contrast-enhanced computed tomography were routinely used to ensure complete removal of HCC.^26,27^ Macroscopic vascular invasion was determined using imaging or histology. Detailed characteristics of the patients enrolled in this study are summarised in Supplementary Table S1.

Patients were followed postoperatively at the outpatient clinic with regular surveillance for recurrence using the serum alpha-fetoprotein level, abdominal ultrasonography, and chest radiography, at 2–4-month intervals.^26–28^ When tumour recurrence or metastasis was suspected, further examinations, including computed tomography and hepatic angiography, were performed. Patients with confirmed recurrence received further treatment, including a second surgical resection, transarterial chemoembolisation, radiofrequency ablation, or percutaneous ethanol injection. The median follow-up was 52.7 months (range 1–132 months). Of the 453 patients who were examined during the follow-up period, 207 patients (45.7%) died, 263 patients (58.1%) were diagnosed with tumour recurrence, and 144 patients (31.8%) remained alive without recurrence. Relapse-free survival (RFS) was defined as the interval between surgery and the first of recurrence or death, or between surgery and the last observation for patients without recurrence. OS was defined as the interval between surgery and death or between surgery and the last observation for surviving patients.

### Tissue microarray construction

Tissues were used to construct a tissue microarray, as described previously.^26^ Briefly, tissue blocks containing the advancing edges of tumoural HCC tissue were used for tissue microarray (TMA) construction. Duplicate 1.0-mm tissue cores were randomly taken from the tumoural area in the paraffin-embedded tissue blocks. TMAs containing the tissue cores were then cut into 4-μm sections for immunohistochemistry (IHC) staining.

### Antibodies

Primary antibodies used in this study were as follows: a mouse anti-human CD68 monoclonal antibody (Dako, Carpinteria, CA, USA), a mouse anti-human CD33 monoclonal antibody (Leica Biosystems, Newcastle, UK), a rabbit anti-human CD11b monoclonal antibody (Abcam, Cambridge, MA, USA), a rabbit anti-human CD3 monoclonal antibody (Thermo Scientific, Rockford, IL, USA), a rabbit anti-human CD20 monoclonal antibody (ZSBio, Beijing, China), a mouse anti-human CD57 monoclonal antibody (ZSBio, Beijing, China), and a rabbit anti-human PD-L1 monoclonal antibody (clone: E1L3N™; Cell Signaling Technology, Danvers, MA, USA).

### Immunohistochemistry

Formalin-fixed, paraffin-embedded tissues were cut into 4-μm thick sections and subjected to immunohistochemical analysis. Sections were dewaxed in xylene and rehydrated through a decreasing ethanol series. To diminish the activity of endogenous peroxidase, sections were placed in 0.3% H_2_O_2_ for 10 min at room temperature. Heat-mediated retrieval was use for antigen retrieval. Slides were incubated overnight at 4 °C with different primary antibodies. Detection was performed using the EnVision Detection Systems (DakoCytomation, Carpinteria, CA, USA) following the manufacturer’s instructions; sections were counter-stained with haematoxylin. Image acquisition was performed using an Eclipse advanced research microscope (Nikon, Melville, NY, USA).

### Multiplex staining and multispectral imaging

To identify the cell subsets expressing PD-L1 in the TME, multiplex immunofluorescence staining was obtained using TSA Plus Fluorescence Kits (PerkinElmer, Foster City, CA, USA) combined with IHC (TSA-IHC). Different primary antibodies were sequentially applied, followed by horseradish peroxidase-conjugated secondary antibody incubation and tyramide signal amplification. The slides were microwave heat-treated after each TSA operation. Nuclei were stained with 4′-6′-diamidino-2-phenylindole (DAPI, Thermo Scientific) after all the human antigens had been labelled.

To obtain multispectral images, the stained slides were scanned using the Vectra System (PerkinElmer), which captures the fluorescent spectra at 20-nm wavelength intervals from 420 to 720 nm with identical exposure time; the scans were combined to build a single stack image.

For colocalisation analysis, images were acquired using a laser confocal microscope (Olympus, Essex, UK) and analysed using FV10-ASW Viewer software (Olympus).

### Image analysis

Images of unstained and single-stained sections were used to extract the spectrum of autofluorescence of tissues and each fluorescein, respectively. The extracted images were further used to establish a spectral library required for multispectral unmixing by Nuance and InForm image analysis software (PerkinElmer). Using this spectral library, we obtained reconstructed images of sections with the autofluorescence removed.

To define PD-L1^+^ tumours, specimens displaying unequivocal membranous or cytoplasmic PD-L1 staining were classified as positive. In the tumour nest, PD-L1-expressing CD68^+^ Mφs showed a membranous staining pattern and were typically seen as variably sized aggregates towards the periphery of the tumour mass, at the edge of necrosis area, or as single cells scattered in the tumour parenchymal area. The percentage of PD-L1^+^ Mφs was estimated as the percentage of total CD68^+^ Mφ, and was scored as negative or positive expression if the percentage was <5% or ≥5%, respectively. PD-L1-expressing TCs with prominent nucleoli and CD68 negative expression typically showed membranous staining and a variable component of cytoplasmic staining. The proportion of PD-L1-positive TCs was evaluated as the percentage of total TCs, and the sample was scored as negative or positive expression if the percentage was <5% or ≥5%, respectively.

### Total RNA isolation and quantitative real-time PCR (qPCR)

Total RNA was extracted from archived frozen tissues collected by Bank of Tumor Resource of Sun Yat-sen University Cancer Center using the TRI Reagent solution (Thermo Scientific) according to the manufacturer’s protocol. The RNA concentration was determined using a nanodrop ND-1000 spectrophotometer (NanoDrop Technologies, Wilmington, DE, USA). Aliquots (2 μg) of total RNA were reverse-transcribed into cDNA using the 5× All-In-One RT MasterMix (Applied Biological Materials Inc, Richmond, BC, Canada). The specific primers used to amplify the genes are listed in Supplementary Table S2. The PCR was performed in triplicate using SYBR Green real-time PCR Master Mix (TOYOBO, Osaka, Japan) in a Roche LightCycler 480 System (Roche Diagnostics, Pleasanton, CA, USA). The cycle threshold (Ct) values did not differ by more than 0.5 among triplicate samples. ΔCt between target genes and the reference gene (*GAPDH*) were calculated to perform the data analysis.

### Microarray assay and data analysis

RNA samples from HCC tumours from 24 cases with different PD-L1 expression patterns defined by multiplex staining were subjected to a microarray assay. Each PD-L1 expression pattern contained six biological repetitions. Sample amplification, labelling, hybridisation onto Agilent whole human genome oligonucleotide microarrays containing 58341 different oligonucleotide probes (SurePrint G3 Human Gene Expression 8 × 60 K Microarray Kit, Agilent), data extraction, and raw data normalisation were performed by Shanghai Biotechnology Corporation (Shanghai, China).

A gene was defined as differentially expressed between different patterns when its mean expression differed by at least twofold between the patterns, with a Student’s *t* tests *p* value < 0.05. The differentially expressed genes (DEGs) were separately analysed for GO term enrichment using the online functional annotation tools DAVID 6.7. Representative enriched GO terms with *p* values < 0.05 were selected from the GO biological process terms in DAVID’s fat database.

To calculate single-phenotype gene set enrichment, Gene Set Enrichment Analysis (GSEA) software was applied to derive the normalised enrichment score (NES) of the gene signatures using the C5 BP (GO biological process) subset collected by the Molecular Signature Database (MSigDB) version 6.1. Differentially enriched core gene sets between different phenotypes were defined by a nominal *p* value < 0.05.

### Statistical analysis

Differences in means for continuous variables were compared using Student’s *t* test or analysis of variance, and differences in proportions were tested by *χ*^2^ test. Kaplan–Meier estimates were calculated and compared using the log-rank test. The Cox proportional hazard regression models were applied to evaluate the prognostic variables for RFS and OS. IBM SPSS (version 21.0; SPSS Inc., Chicago, IL, USA) statistics software was used for all statistical analyses. All data were analysed using two-tailed tests unless otherwise specified, and *p* < 0.05 was considered statistically significant.

## Results

### HCC tissue samples and PD-L1 expression

Our previous studies showed that PD-L1-expressing monocytes/macrophages were highly enriched in the peritumoural stroma of HCC tissues, and could promote tumour progression by impairing T-cell immunity and fostering angiogenesis.^29,30^ Recent studies in other tumour types have revealed that patients with PD-L1 expression on TCs or immune cells in the intra-tumour region showed a better clinical response to anti-PD-1/PD-L1 immunotherapies.^16,21–23^ To fully characterise PD-L1 expression patterns, the clinical significance, and immune status in HCC tumour tissues, 453 intra-tumoural samples from patients with HCC who received curative resection were examined. Most of these patients were male (400/453, 88.3%), and their median age at surgery was 50 years. Among the patients, 91.2% (413/453) were positive for HBV infection; 96.9% (439/453) had Child-Pugh score A; 55.2% (250/453) had a large tumour (>5 cm in diameter), and most patients (335/453, 74.0%) had single tumour foci. Vascular invasion was observed in 11.9% (54/453) of cases. There were 68.7% (311/453) and 31.3% (142/453) patients at stage I and II, and stage III and IV, respectively, according to the TNM staging system (7th Edition). In addition, the histological grade of tumour differentiation was defined as I and II (59.8%, 271/453), and III and IV (40.2%, 182/453) according to the Edmondson grading system. The detailed clinical and pathological features of the series are summarised in Supplementary Table S1.

**Fig. 1:**
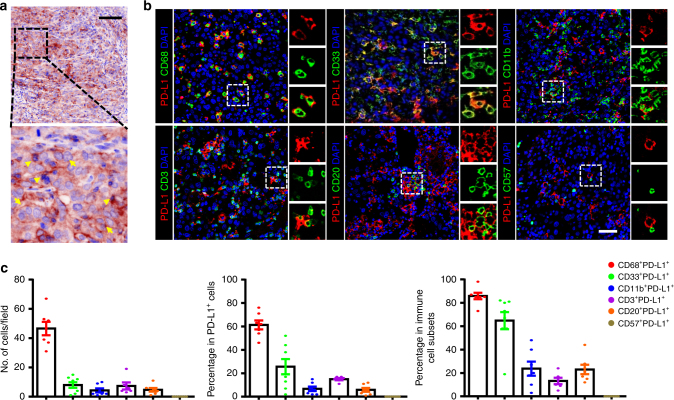
PD-L1 expression in HCC tumour tissues. **a** Representative image of PD-L1 (brown) by IHC-TSA staining in HCC tumour tissue. Arrows indicate representative PD-L1^+^ tumour cells, triangles indicate representative PD-L1^+^ immune-cell-like cells. Scale bar, 100 μm. **b** Multiplex immunofluorescence staining with the indicated immune cell markers in HCC tumour tissue. PD-L1 staining is shown in red; markers of immune cell markers in green; and DAPI staining in blue. Scale bar, 50 μm. **c** Analysis of the percentages of double-positive immune cells in total PD-L1^+^ cells (middle), counts of double-positive cells per field (left), and the percentage of double-positive immune cells within different immune cell subsets (right). Data are expressed as the mean ± SEM

Staining of the PD-L1 protein in HCC tissues was performed using TSA-IHC and analysed using the inForm system. Membranous PD-L1 expression could be found on both TCs and immune cells in the intra-tumoural region of HCC tissues (Fig. 1a). Of the 453 samples examined, most samples were weak or negative for PD-L1 expression, only 29.8% (135/453) of the samples were positively stained for PD-L1 expression on TCs or non-TCs. To identify the cell types of the PD-L1^+^ immune cells, we performed colocalisation studies and calculated the proportion of PD-L1^+^ cells within each cellular subset in samples of PD-L1^+^ immune cells (Fig. 1b). The majority of PD-L1-expressing cells in these samples (*n* = 11) were CD68^+^ myeloid cells, representing 61.29 ± 3.84 % of total PD-L1^+^ cells. T cells and B cells represented only a minority of all PD-L1^+^ cells in HCC tumour tissues (15.0 ± 0.93% and 5.86 ± 1.63%, respectively; Fig. 1c); PD-L1^+^ NK cells were barely detected in these samples. In addition, we noted that PD-L1^+^ cells could also express CD33 (25.63 ± 6.50%), but rarely expressed CD11b (6.71 ± 1.92%), which indicated that PD-L1 could be expressed on different subpopulations of myeloid cells in HCC. Collectively, these data indicated that TCs and CD68^+^ macrophages were the primary PD-L1-expressing cell types in the intra-tumour area of HCC tissues.

### Different expression patterns of PD-L1 in HCC tumours

**Fig. 2:**
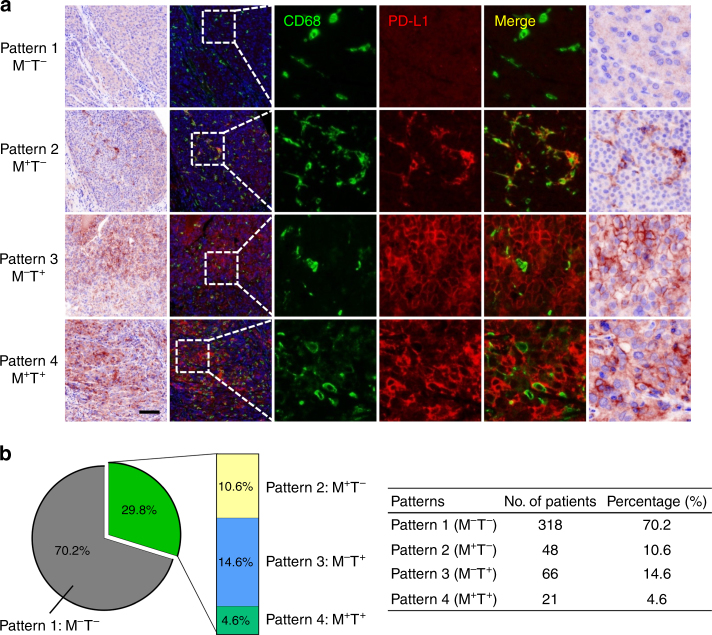
Different expression patterns of PD-L1 in HCC tumours. **a** Paraffin-embedded HCC tumour tissue sections with PD-L1 expression Pattern 1 (M^–^T^–^), Pattern 2 (M^+^T^–^), Pattern 3 (M^–^T^+^), and Pattern 4 (M^+^T^+^) were subjected to TSA-IHC using anti-human CD68 and anti-human PD-L1 antibodies. Images were obtained using the Vectra System and analysed by inForm image analysis software. Images presented in bright field are in the first column, with corresponding fluorescent images in the second column. Small-scale images are shown in the following columns. Scale bar, 100 μm. **b** Graphs showing the number of patients and the percentage of each pattern

Recent studies have proposed that different expression patterns of PD-L1 might lead to different biological significance and clinical benefits of anti-PD-1/PD-L1 immune therapies.^16,17,24,31,32^ Therefore, we performed double-colour immunofluorescent staining of PD-L1 with CD68, which could mark most PD-L1^+^ immune cells in HCC, and divided samples into four groups according to their PD-L1 expression patterns in the intra-tumoural region (Fig. 2a): (1) Absence of PD-L1 expression on both Mφs (M) and TCs (T; Pattern 1: M^–^T^–^); (2) only Mφs express PD-L1 (Pattern 2: M^+^T^–^); (3) only TCs express PD-L1 (Pattern 3: M^–^T^+^); and (4) PD-L1 expression on both Mφs and TCs (Pattern 4: M^+^T^+^). We then analysed the proportions of the different expression patterns of PD-L1 in the 453 patients with HCC treated by resection (Supplementary Table S1). The incidence rates of PD-L1 expression on Mφ or TC were 15.2% (Pattern 2 and 4: M^+^T^–^ and M^+^T^+^) and 19.2% (Pattern 3 and 4: M^–^T^+^ and M^+^T^+^), respectively. Only 4.6% (21/453) of the patients had tumours with PD-L1 expression on both Mφs and TCs (Pattern 4: M^+^T^+^), while 10.6% (48/453; Pattern 2: M^+^T^–^) and 14.6% (66/453; Pattern 3: M^–^T^+^) of patients had PD-L1 expression only on Mφs or TCs, respectively (Fig. 2b).

### Differential impact of PD-L1 expression patterns on disease progression in HCC patients

**Fig. 3:**
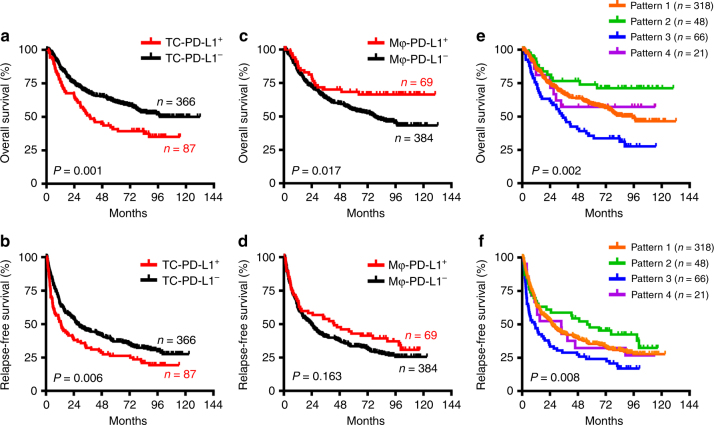
Differential impact of PD-L1 expression patterns on disease progression in patients with HCC. Cumulative OS (**a**, **c**, **e**) and RFS (**b**, **d**, **f**) of TC-PD-L1 expression (**a**, **b**), Mφ-PD-L1 expression (**c**, **d**), and combined expression patterns (**e**, **f**), were calculated by the Kaplan–Meier method and analysed using the log-rank test

To evaluate the differential impact of the PD-L1 expression patterns on disease progression in patients with HCC, the correlations between the PD-L1 expression patterns and patients’ survival were investigated. Kaplan–Meier survival analysis revealed a striking negative association between the presence of PD-L1-expressing TCs and patients’ OS and RFS, respectively (*p* = 0.001 and 0.006 for OS and RFS, respectively; Fig. 3a, b). Interestingly, the density of PD-L1-expressing Mφs was positively correlated with patients’ OS (*p* = 0.017; Fig. 3c). Survival analysis showed that patients with the Mφ-PD-L1^+^ but TC-PD-L1^−^ expression pattern (Pattern 2) had the longest OS (median not reached; 5-year OS, 74.0%; Fig. 3e) and RFS (median, 56.0 months; 5-year RFS, 48.0%; Fig. 3f); whereas patients with the Mφ-PD-L1^−^ but TC-PD-L1^+^ expression pattern (Pattern 3) had the shortest OS (median, 35.1 months; 5-year OS, 35.0%; Fig. 3e) and RFS (median, 10.7 months; 5-year RFS, 24.0%; Fig. 3f).

The presence of PD-L1-expressing Mφs was significantly associated with tumour differentiation (*p* = 0.003; Supplementary Table S3). Multivariate analysis revealed that PD-L1 expression on TCs or Mφs were both independent prognostic factors for OS (HR = 1.168, *p* = 0.004 for TC-PD-L1; HR = 0.708, *p* = 0.003 for Mφ-PD-L1; Table 1). Taken together, these results suggested that the presence of PD-L1 on TCs correlates with tumour progression, whereas the expression of PD-L1 on Mφs has a protective role for the prognosis of patients with HCC.

**Table 1:** Univariate and multivariate analysis of factors associated with overall survival and relapse-free survival

Variables	OS				RFS			
	Univariate	Multivariate			Univariate	Multivariate		
	*p*	HR	95% CI	*p*	*p*	HR	95% CI	*p*
Model 1								
Age, y (>50/≤50)	0.976				0.289			
HBsAg (present/absent)	0.883				0.047	1.649	1.057–2.572	0.028
AFP, ng/mL (>25/≤25)	<0.0001	1.536	1.126–2.095	0.007	0.002	1.277	1.004–1.625	0.046
Tumour size, cm (>5/≤5)	0.001	1.04	0.765–1.414	0.801	0.006	1.062	0.831–1.358	0.631
Tumour number (multiple/single)	<0.0001	1.329	0.928–1.903	0.121	<0.0001	1.28	0.938–1.746	0.119
Vascular invasion (present/absent)	<0.0001	3.342	2.261–4.938	<0.0001	<0.0001	2.084	1.471–2.952	<0.0001
TNM stage (III+IV/I+II)	<0.0001	1.729	1.18–2.535	0.005	<0.0001	1.394	1.008–1.928	0.045
Differentiation (III+IV/I+II)	0.447				0.512			
TC-PD-L1 (positive/negative)	0.001	1.168	1.052–1.297	0.004	0.006	1.142	1.044–1.25	0.004
Model 2								
Age, y (>50/≤50)	0.976				0.289			
HBsAg (present/absent)	0.883				0.047	1.575	1.011–2.456	0.045
AFP, ng/mL (>25/≤25)	<0.0001	1.623	1.187–2.22	0.002	0.002	1.304	1.023–1.664	0.032
Tumour size, cm (>5/≤5)	0.001	1.063	0.781–1.447	0.699	0.006	1.081	0.845–1.383	0.535
Tumour number (multiple/single)	<0.0001	1.257	0.875–1.805	0.217	<0.0001	1.246	0.911–1.705	0.169
Vascular invasion (present/absent)	<0.0001	3.713	2.494–5.528	<0.0001	<0.0001	2.223	1.561–3.164	<0.0001
TNM stage (III+IV/I+II)	<0.0001	1.662	1.125–2.455	0.011	<0.0001	1.34	0.965–1.86	0.081
Differentiation (III+IV/I+II)	0.447				0.512			
Mφ-PD-L1 (positive/negative)	0.018	0.708	0.562–0.891	0.003	0.165	0.872	0.739–1.03	0.107

Variables associated with overall survival or relapse-free survival by univariate analysis were adopted as covariates in multivariate analysis and entered into the equation by the forward selection based on likelihood ratio test.

*OS* overall survival, *RFS* relapse-free survival, *HR* hazard ratio, *CI* confidence interval, *TC* tumour cells, *Mφ* macrophage

### Correlation of PD-L1 expression patterns and immune status of the TME

**Fig. 4:**
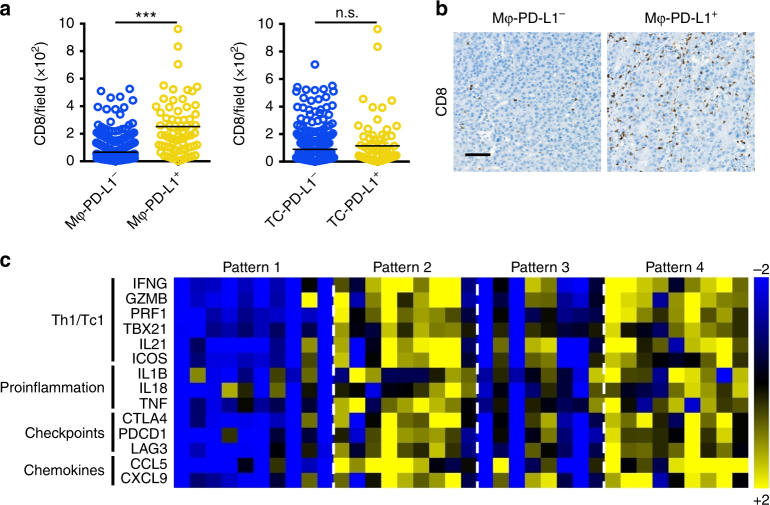
PD-L1 expression on Mφs correlated with an active immune microenvironment. **a** The density of tumour-infiltrating CD8^+^ T cells in Mφ-PD-L1^−^ tumours and Mφ-PD-L1^+^ tumours (left), and in TC-PD-L1^−^ tumours and TC-PD-L1^+^ tumours (right). ***p < 0.001; n.s. not significant. **b** Representative images of IHC staining of CD8 in sections with Mφ-PD-L1^−^ and Mφ-PD-L1^+^ HCC tumour tissue. Images are at ×200 magnification. Scale bar, 100 μm. **c** Heatmap showing immune-related gene expression profiles in tumour samples with different PD-L1 expression patterns. The expression levels of different genes were assessed using quantitative real-time PCR. Sets of genes were defined by functional relevance (Th1/Tc1, proinflammation, immune checkpoints, and chemokines)

The expression of PD-L1 (B7-H1) is often induced or maintained by inflammatory cytokines.^29,33,34^ Thus, the upregulation of PD-L1 expression in tumour tissues could be viewed as a reflection of endogenous inflammatory immune responses, but not simply as the dominance of immune suppression. It has been proposed that, based on having either relatively high or low immune cell infiltration, tumours may be immunologically classified into ‘hot’ (inflamed) or ‘cold’ (non-inflamed) phenotypes, respectively.^35–37^ Accordingly, clinical evidences showed that patients with an immunogenic TME had a better clinical response to anti-PD-1/PD-L1 immunotherapies.^16^ Therefore, we first examined CD8^+^ T-cell infiltration in HCC tumours with different PD-L1 expression patterns. As shown in Fig. 4a, b, the density of CD8^+^ T cells was significantly higher in tumours with Mφ-PD-L1^+^ compared with that in Mφ-PD-L1^−^ tumours (*p* < 0.0001); whereas there was no correlation between TC-PD-L1 expression and CD8^+^ T-cell infiltration. We further examined the expression of immune response-related genes using quantitative real-time polymerase reaction (qPCR). As expected, Mφ-PD-L1^+^ tumours displayed a high level of active immune response-related gene expression (Fig. 4c), including *IFNG*, *GZMB*, and *PRF1*.

**Fig. 5:**
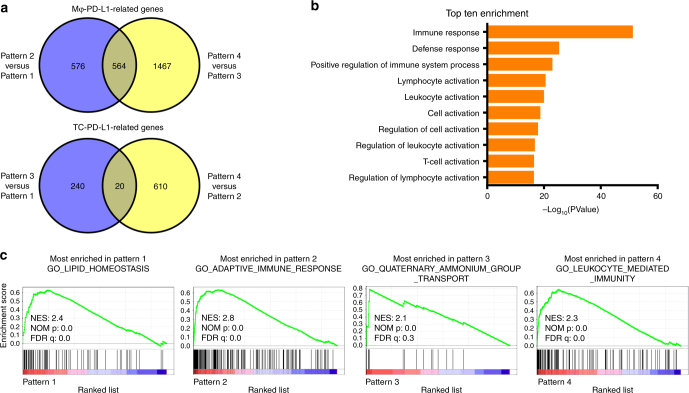
PD-L1 expression patterns correlated with different TMEs. **a** Venn diagram analysis of differentially expressed genes between different PD-L1 expression patterns. **b** Top ten GO terms from the GO enrichment analysis using the common differentially expressed genes between Pattern 2 versus Pattern 1 and Pattern 4 versus Pattern 3. **c** Gene Set Enrichment Analysis using microarray data of one arbitrary PD-L1 expression pattern versus the other patterns. Selected gene sets are shown. NES normalised enrichment score, NOM p nominal *p* value, FDR q false discovery rate

To further characterise the immune status of the TME in the HCC tissues with differential PD-L1 expression patterns, we performed gene expression microarray analysis for 24 tumour samples with different PD-L1 expression patterns. Globally, there were 1140 DEGs in the comparison between Pattern 2 and Pattern 1, and 2031 DEGs when comparing Pattern 4 with Pattern 3 (Supplementary Table S4). Venn diagram analysis revealed that 564 genes were commonly up- or downregulated across these groups, which represented Mφ-PD-L1-related genes (Fig. 5a, top and Supplementary Table S5). These genes were further corroborated by Gene Ontology (GO) analysis, which showed that GO terms for antigen processing and presentation, leucocyte activation, and immune effector process were enriched (Fig. 5b and Supplementary Table S6). Whereas in the analysis of the DEGs between Pattern 3 and Pattern 1, and in Pattern 4 compared with Pattern 2, only 20 common genes were identified, which represented TC-PD-L1-related genes (Fig. 5a, bottom and Supplementary Table S5). Among these 20 common genes, the expression levels of *CDK20*, *AKAP7*, *AZIN2*, *LIX1L*, *OSCP1*, and *SLFN12* were upregulated, while *HLF* was downregulated in TC-PD-L1^+^ tumours (Pattern 3 and Pattern 4).

To determine the differences in cellular process and functional states in HCC tumours with different PD-L1 expression patterns, we performed GSEA using the C5 GO biological process collections in MSigDB. As shown in Fig. 5c, lipid homeostasis, adaptive immune response, quaternary ammonium group transport, and leucocyte-mediated immunity were the most enriched gene sets in Patterns 1–4, respectively (Fig. 5c and Supplementary Table S7 and S8). These data suggested that the identified PD-L1 expression patterns correlated with diverse tumour functional states, and, correspondingly, different TMEs. Taken together, these data suggested that the HCC tissues with Mφ-PD-L1^+^ patterns correlated with a ‘hot’ TME, with higher numbers of infiltrating CD8^+^ T cells and higher levels of immune-related gene expression. This identified PD-L1 expression pattern might serve as a novel method to depict PD-L1 expression to predict the therapeutic responsiveness of anti-PD-1/PD-L1 immunotherapies.

## Discussion

Immune checkpoint therapies targeting PD-1 and PD-L1 have achieved remarkable clinical responses across different tumour types, including HCC; however, the objective response rates varied. Accumulating data have suggested that intra-tumoural expression of PD-L1 could be a potential biomarker to predict the clinical response to anti-PD-1/PD-L1 therapies in patients with melanoma, non-small-cell lung carcinoma, and renal cell carcinoma.^12,14,21,38,39^ To characterise and investigate PD-L1 expression in HCC in detail, we performed double staining using TSA-IHC in 453 patients with HCC. Our results showed that TCs and Mφs were the primary PD-L1-expressing cell types in the intra-tumour area of HCC tissues. By TSA-IHC staining of PD-L1 and CD68, we divided patients into four expression patterns: (1) M^–^T^–^; (2) M^+^T^–^; (3) M^–^T^+^; and (4) M^+^T^+^ according to the expression of PD-L1 on TCs and Mφs. Survival analysis showed the presence of PD-L1 on TCs correlated with tumour progression, whereas the expression of PD-L1 on Mφs had a protective role for the prognosis of patients with HCC. Moreover, qPCR and microarray data revealed that Mφ-PD-L1^+^ tumours exhibited an activated immune microenvironment, with high numbers of infiltrating CD8^+^ T cells and high levels of immune-related gene expression.

Tumour responses to anti-PD-1/PD-L1 immunotherapy are mediated by T cells that had been previously blocked by the PD-1−PD-L1 interaction.^40–42^ Thus, it is reasonable to assume that pre-existing tumour-infiltrating lymphocytes and PD-L1 expression might correlate with clinical response to anti-PD-1/PD-L1 immunotherapy. Clinical trials across different tumour types have confirmed this hypothesis, and the US Food and Drug Administration has approved several IHC test assays to detect PD-L1, which may support clinical decisions for anti-PD-1/PD-L1 therapies. PD-L1 can be expressed on both TCs and immune cells, which have different biological and clinical significance; however, the currently approved assays require a well-trained pathologist to determine the positive cell type and expression level. A recent review of analytical assays reported that a lack of pre-specified criteria could lead to poor intra- and inter-observer concordance in the assessment of PD-L1 expression on immune cells.^43^ In the present study, we found that CD68 could mark most PD-L1^+^ immune cells in HCC tumours, and that TSA-IHC for CD68 and PD-L1 double staining could clearly distinguish PD-L1 expression on TCs and immune cells. These results provided a novel method to determine PD-L1 expression and have a potential application in patient selection for anti-PD-1/PD-L1 therapies.

Previous studies have reported the functional roles of PD-L1 expression on T cells and B cells, such as mediating DC maturation by naïve T cells, increasing the survival of effector T cells, and regulating humoral immunity by the interaction between Breg cells with T_FR_ cells.^44–46^ However, the clinical significance of these PD-L1^+^ lymphocytes is currently unknown. Recent studies revealed the essential role of the expression of PD-L1, especially on myeloid cells, in determining the efficacy of PD-L1 blockade.^24,47^ Thus, the evaluation of Mφ-PD-L1^+^ tumours should have an important biological implication. In the present study, we showed that CD68^+^ myeloid cells were the major PD-L1^+^ immune cells in HCC (61.29 ± 3.84 % of total PD-L1^+^ cells), and were further used to divide the samples into four groups with different PD-L1 expression patterns.

Several groups have attempted to assess the relationship between PD-L1 expression and patients’ outcome in HCC; however, these studies mostly examined the overall expression of PD-L1, or simply classified PD-L1 expression on TCs or immune cells by IHC staining.^48,49^ In the present study, CD68 and PD-L1 double staining was applied to determine the positive cell types for PD-L1. Consistent with other reports, TC-PD-L1 expression correlated with poor outcome in patients with HCC.^48^ Interestingly, we found that Mφ-PD-L1 expression was associated with good prognosis in these patients.

Our data further showed that Mφ-PD-L1^+^ tumours exhibited an activated TME, with high numbers of infiltrating CD8^+^ T cells and high levels of immune-related gene expression, including leucocytes chemotaxis, Th1/Tc1 active immune responses, inflammatory cytokines, and immune checkpoints. Microarray analysis confirmed that Mφ-PD-L1^+^ tumours displayed high expression of genes engaged in the immune response and lymphocyte activation. Thus, the good prognostic value of Mφ-PD-L1 might be because of the active anti-tumoural TME of the HCC tumours. However, it remains to be determined whether the therapeutic efficacy of PD pathway blockade is similar between patients with Mφ-PD-L1^+^ tumours and TC-PD-L1^+^ tumours in HCC.

Several factors that influence the constitutive PD-L1 expression on TCs have been proposed, such as EGFR activation, PTEN deletions, the MLL1-H3K4me3 axis, and CDK5 disruption.^50–53^ In the present study, we showed that TC-PD-L1 expression did not correlate with T-cell infiltration or the levels of immune response genes. Differential gene expression analysis using a microarray revealed that several genes were commonly upregulated among TC-related genes, such as *CDK20*. A recent study showed that combined CDK20 inhibition with immune checkpoint blockade might provide more efficient therapeutic effects for patients with HCC.^54^ However, the relationship and underlying mechanism of the association of these genes with PD-L1 expression in HCC is unclear and requires further investigation.

In summary, the expression pattern of PD-L1, as determined by CD68 and PD-L1 double staining, could serve as an important prognostic factor in HCC. This technically simple method could provide a novel approach to detect PD-L1 expression, which might help identify patients who may benefit from anti-PD-1/PD-L1 immunotherapies.

## Electronic supplementary material

Supplementary Table S1

Supplementary Table S2

Supplementary Table S3

Supplementary Table S4

Supplementary Table S5

Supplementary Table S6

Supplementary Table S7

Supplementary Table S8

## Availability of data and material

The authenticity of this article has been validated by uploading the key raw data onto the Research Data Deposit public platform (www.researchdata.org.cn), with the pproval RDD number as RDDB2018000319.
